# Proton Pump Inhibitor-Associated Restless Legs Syndrome

**DOI:** 10.7759/cureus.113554

**Published:** 2026-07-28

**Authors:** Susan D Leonard, Albert K Bui

**Affiliations:** 1 Geriatric Medicine, University of California, Los Angeles (UCLA) Medical Center, Los Angeles, USA; 2 Internal Medicine and Geriatrics, Colima Medical Clinic, Los Angeles, USA

**Keywords:** adverse drug reaction, pantoprazole, proton pump inhibitor, restless leg syndrome, sleep disturbances

## Abstract

Proton pump inhibitors (PPIs) are among the most commonly prescribed medications worldwide and are generally well tolerated. Although gastrointestinal adverse effects are well recognized, sleep-related adverse effects are considered uncommon and remain underrecognized. We present the case of a 41-year-old man who developed restless legs syndrome resulting in significant sleep disturbance shortly after initiation of pantoprazole therapy for nonsteroidal anti-inflammatory drug (NSAID)-induced duodenal ulcer bleeding. Despite empiric oral iron supplementation for suspected iron deficiency following acute gastrointestinal blood loss, his symptoms persisted throughout the eight-week course of therapy and resolved within several days after discontinuation of pantoprazole. Although iron deficiency represented an important alternative explanation, the close temporal relationship with pantoprazole therapy, lack of response to iron supplementation, and rapid symptom resolution following drug discontinuation suggest a probable medication-associated adverse effect. This case highlights an uncommon but potentially reversible adverse drug reaction and reinforces the importance of considering PPIs in the differential diagnosis of new-onset restless legs syndrome or sleep disturbances after initiation of therapy.

## Introduction

Proton pump inhibitors (PPIs) are the cornerstone of therapy for acid-related disorders, including gastroesophageal reflux disease (GERD), peptic ulcer disease (PUD), and duodenal ulcer disease [1,2]. They provide superior symptom relief, promote mucosal healing, and reduce disease recurrence and complications, making them among the most commonly prescribed medications worldwide [1-3]. PPIs are generally well tolerated, with the most frequently reported adverse effects including headache, nausea, vomiting, and diarrhea [3]. Sleep-related adverse effects are considered uncommon, occurring in fewer than 1% of patients in clinical trials and Food and Drug Administration (FDA) labeling [4]. In fact, PPIs are generally expected to improve rather than worsen sleep quality because effective treatment of GERD reduces nocturnal reflux symptoms, a major contributor to sleep disruption among patients with GERD [5-7]. However, emerging pharmacovigilance and observational studies suggest that sleep-related adverse effects, including insomnia, restless legs syndrome, and rapid eye movement (REM) sleep behavior disorder, may be underrecognized [8-10]. Here, we report the case of a 41-year-old man who developed reversible restless legs syndrome resulting in significant sleep disturbance following initiation of pantoprazole therapy.

## Case presentation

A 41-year-old man without significant past medical history presented to the emergency department with hypotension, tachycardia, and melena. He reported taking over-the-counter nonsteroidal anti-inflammatory drugs (NSAIDs) for three days several weeks earlier to treat myalgias and headaches associated with a viral upper respiratory infection. Baseline outpatient laboratory studies obtained three weeks before hospitalization were unremarkable (Table 1).

**Table 1:** Baseline outpatient laboratory findings (3 weeks before hospitalization) eGFR: estimated glomerular filtration rate

Test Category	Parameter	Result	Normal Range
Hematology	Hemoglobin	16.2 g/dL	13.2 - 17.1 g/dL
	Mean corpuscular volume	89.5 fL	81.4 - 101.7 fL
	Red cell distribution width	12.6 %	11.0 - 15.0 %
Renal function	Sodium	140 mmol/L	135 - 146 mmol/L
	Potassium	4.2 mmol/L	3.5 - 5.3 mmol/L
	Urea	14 mg/dL	7 - 25 mg/dL
	Creatinine	0.97 mg/dL	0.6 - 1.29 mg/dL
	eGFR	101 mL/min/1.73 m²	>60 mL/min/1.73 m²
Liver profile	Alkaline phosphatase	96 U/L	36 - 130 U/L
	Aspartate aminotransferase	35 U/L	10 - 40 U/L

On presentation, his heart rate was 120 beats/min (baseline 60 beats/min), and his blood pressure was 100/60 mmHg (baseline 120/80 mmHg). Stool guaiac testing was positive. Admission laboratory evaluation demonstrated acute blood loss anemia with a progressive decline in hemoglobin on serial measurements, while the remainder of his laboratory studies, including coagulation profile, renal function, liver function, and lipase, were unremarkable (Table 2). He received an 80-mg intravenous pantoprazole bolus followed by a continuous infusion at 8 mg/hour.

**Table 2:** Hospital admission laboratory findings INR: international normalized ratio; eGFR: estimated glomerular filtration rate

Test Category	Parameter	Result	Normal Range
Hematology	Hemoglobin (time 0)	12.6 g/dL	13.2 - 17.1 g/dL
	Hemoglobin (6 hours later)	11.4 g/dL	13.2 - 17.1 g/dL
	Hemoglobin (12 hours later)	10.6 g/dL	13.2 - 17.1 g/dL
Coagulation profile	Prothrombin time	13.9 sec	11.6-14.2 sec
	Partial thromboplastin time	30 sec	24.0-35.0 sec
	INR	1.03	0.90-1.10
Renal function	Urea	27 mg/dL	9-23 mg/dL
	Creatinine	0.83 mg/dL	0.6-1.1 mg/dL
	eGFR	113 mL/min/1.73 m²	>59 mL/min/1.73 m²
Liver profile	Alkaline phosphatase	67 U/L	46-116 U/L
	Aspartate aminotransferase	18 U/L	13-40 U/L
	Lipase	44 U/L	12-53 U/L

Esophagogastroduodenoscopy (EGD) performed the following day revealed two clean-based duodenal ulcers without active bleeding. Biopsies for *Helicobacter pylori* were negative. He was discharged on oral pantoprazole twice daily for an eight-week course. 

At outpatient follow-up, his vital signs had returned near baseline. During treatment with pantoprazole, he developed severe nightly symptoms consistent with restless legs syndrome that significantly disrupted his sleep. Given his recent gastrointestinal blood loss, iron deficiency was suspected, and empiric oral iron supplementation was initiated without symptomatic improvement. Follow-up laboratory evaluation after completion of the eight-week course of pantoprazole confirmed iron deficiency anemia despite oral iron supplementation (Table 3). Nevertheless, his restless legs symptoms resolved within several days after completing pantoprazole therapy. He reported no prior history of restless legs syndrome or chronic sleep disturbance before this episode.

**Table 3:** Laboratory findings 8 weeks post-hospitalization

Test Category	Parameter	Result	Normal Range
Hematology	Hemoglobin	9.2 g/dL	13.2 - 17.1 g/dL
	Mean corpuscular volume	72.9 fL	81.4 - 101.7 fL
	Red cell distribution width	19.7 %	11.0 - 15.0 %
Iron studies	Total iron	14 mcg/dL	50 - 180 mcg/dL
	Total iron binding capacity	515 mcg/dL	250 - 425 mcg/dL
	Iron saturation	3 %	20 - 48 %
	Ferritin	4 ng/mL	38 - 380 ng/mL

## Discussion

PPI-related sleep disturbances are an uncommon adverse effect and were seen in <1% of FDA clinical trials. In fact, PPIs are generally thought to improve rather than worsen sleep quality because effective treatment of GERD reduces nocturnal reflux symptoms, a major contributor to sleep disruption among patients with GERD [5-7]. However, in the FDA Adverse Event Monitoring System (AEMS), which is a publicly accessible database containing adverse event reports and complaints submitted to the FDA in post-marketing safety, a higher percentage of psychiatric adverse effects, including sleep-related disorders, were reported in 12.83% of all adverse event reports [8-9]. These studies included reports of insomnia, REM sleep behavior disorder, sleep terrors, and sleep disorders involving several PPIs such as esomeprazole, omeprazole, lansoprazole, and pantoprazole. Although spontaneous reporting systems cannot establish causality because of reporting bias, they are particularly valuable for identifying uncommon adverse drug reactions that may not be detected during clinical trials. The consistency of reports across several agents suggests these adverse effects may represent a class effect rather than being unique to a single PPI. Our case highlights that although uncommon, PPI-associated sleep disturbances should remain in the differential diagnosis when symptoms develop shortly after medication initiation and resolve following discontinuation.

In a large observational study of over 63,000 participants, PPI use was associated with restless legs syndrome (RLS) with an odds ratio of 1.43 [10]. Interestingly, while RLS is thought to be caused in part by low cellular iron levels [11-13], this study found PPI-associated RLS was independent of iron levels. These findings suggest that impaired iron absorption alone may not fully explain PPI-associated RLS and that additional mechanisms are likely involved.

Several mechanisms have been proposed to explain PPI-associated sleep disturbances. PPIs may exert central nervous system (CNS) effects through limited penetration of the blood-brain barrier, although the clinical significance of this remains uncertain [14]. Experimental animal studies have demonstrated that lansoprazole suppresses neuronal activity within the locus coeruleus, a brainstem nucleus involved in regulation of arousal and REM sleep, resulting in increased REM sleep and altered sleep architecture [15]. Additionally, in vitro and animal studies have shown that omeprazole inhibits tryptophan hydroxylase (TPH1 and TPH2), the rate-limiting enzymes involved in serotonin synthesis, providing another potential mechanism through which PPIs may influence sleep-wake regulation [16]. Although these mechanisms have not been fully established in humans, they provide biologic plausibility for the spectrum of sleep-related adverse events reported with PPIs.

Our patient's clinical course demonstrated a clear temporal relationship with pantoprazole therapy. He developed severe nocturnal symptoms consistent with restless legs syndrome shortly after initiation of high-dose PPI therapy, experienced no meaningful improvement despite empiric oral iron supplementation, and reported rapid symptom resolution within several days after completing the planned eight-week course of treatment. Although his approximately 6 g/dL decline in hemoglobin following upper gastrointestinal bleeding likely resulted in significant iron depletion and represents an important alternative explanation, pantoprazole-induced gastric acid suppression may also have impaired absorption of oral non-heme iron, thereby limiting the response to iron supplementation. Follow-up laboratory evaluation eight weeks after hospitalization demonstrated persistent iron deficiency anemia despite oral iron supplementation (Table 3). Nevertheless, his restless legs symptoms resolved within several days after discontinuation of pantoprazole, a timeline that would not be expected from iron repletion alone. Furthermore, observational evidence suggesting that PPI-associated restless legs syndrome may occur independently of iron status [10] supports the possibility that pantoprazole served as the primary trigger, with acute blood loss potentially increasing susceptibility. Taken together, the temporal relationship between drug exposure and symptom onset, persistence despite iron supplementation, prompt resolution following drug discontinuation, absence of a prior history of restless legs syndrome, and supporting evidence from the literature favor a probable medication-associated adverse effect rather than iron deficiency alone.

This case has several important clinical implications. PPIs remain among the most commonly prescribed medications worldwide, and many patients continue therapy for prolonged periods. Clinicians evaluating patients with new-onset insomnia, nocturnal restlessness, or restless legs symptoms may first consider primary sleep disorders, neurologic disease, or psychiatric conditions while overlooking medication-induced causes. Greater awareness of PPI-associated sleep disturbances may help clinicians recognize this uncommon but potentially reversible adverse effect, avoid unnecessary diagnostic evaluations, and consider a trial of medication discontinuation or alternative acid-suppressive therapy when clinically appropriate.

## Conclusions

This case highlights a potential association between proton pump inhibitor (PPI) therapy and reversible restless legs syndrome resulting in significant sleep disturbance. Although uncommon, PPI-associated sleep disturbances have been increasingly recognized in pharmacovigilance and observational studies. Clinicians should consider PPIs in the differential diagnosis of new-onset sleep disturbances or restless legs syndrome following initiation of therapy, particularly when symptoms improve after medication discontinuation. Greater awareness of this uncommon but potentially reversible adverse drug reaction may facilitate earlier recognition, avoid unnecessary diagnostic evaluations, and guide appropriate management.
